# Transgenic mouse model for imaging of ATF4 translational activation-related cellular stress responses *in vivo*

**DOI:** 10.1038/srep46230

**Published:** 2017-04-07

**Authors:** Takao Iwawaki, Ryoko Akai, Takae Toyoshima, Naoki Takeda, Tomo-o Ishikawa, Ken-ichi Yamamura

**Affiliations:** 1 Division of Cell Medicine, Department of Life Science, Medical Research Institute, Kanazawa Medical University, 1-1 Daigaku, Uchinada, Kahoku, Ishikawa 920-0293, Japan; 2 Iwawaki laboratory, Education and Research Support Center, Graduate School of Medicine, Gunma University, 3-39-22 Showa-machi, Maebashi, Gunma 371-8511, Japan; 3 Division of Developmental Genetics, Institute of Resource Development and Analysis, Kumamoto University, 2-2-1 Honjo, Kumamoto 860-0811, Japan; 4 TransGenic Inc, 7-1-14 Minatojima-minamimachi, Chuo-ku, Kobe, Hyogo 650-0047, Japan; 5 Yamamura Project Laboratory, Institute of Resource Development and Analysis, Kumamoto University, 2-2-1 Honjo, Chuoku, Kumamoto 860-0811, Japan

## Abstract

Activating transcription factor 4 (ATF4) is a translationally activated protein that plays a role in cellular adaptation to several stresses. Because these stresses are associated with various diseases, the translational control of ATF4 needs to be evaluated from the physiological and pathological points of view. We have developed a transgenic mouse model to monitor the translational activation of ATF4 in response to cellular stress. By using this mouse model, we were able to detect nutrient starvation response, antivirus response, endoplasmic reticulum (ER) stress response, and oxidative stress *in vitro* and *ex vivo*, as well as *in vivo*. The reporter system introduced into our mouse model was also shown to work in a stress intensity-dependent manner and a stress duration-dependent manner. The mouse model is therefore a useful tool for imaging ATF4 translational activation at various levels, from cell cultures to whole bodies, and it has a range of useful applications in investigations on the physiological and pathological roles of ATF4-related stress and in the development of clinical drugs for treating ATF4-associated diseases.

ATF4 (also known as C/ATF, CREB-2, mTR67, or TAXCREB67) belongs to the activating transcription factor (ATF)/cyclic AMP responsive element binding (CREB) family of proteins, which contains a basic leucine zipper domain and recognizes the consensus DNA sequences TGACGT(C/A)(G/A)^1^. The molecular function of mammalian ATF4 is mainly in controlling the expression of several specific genes associated with stress response, homeostasis, development, fertility, and memory^2^. ATF4 mRNA is expressed in all tissues examined so far^3^,^4^,^5^; however, its expression levels are regulated by a variety of extracellular signals in different cell types^6^,^7^,^8^,^9^,^10^,^11^,^12^. The expression level of ATF4 protein is also regulated by two mechanisms: translational control and stability control. ATF4 stability is modulated by the SCF^βTrCP^ class of ubiquitin ligase^13^ and by histone acetyl-transferase p300^14^. ATF4 translation is induced by phosphorylation of the eukaryotic initiation factor 2α (eIF2α) as follows. ATF4 mRNA contains two or three upstream open reading frames (uORFs), so that a low level of phosphorylated eIF2α leads to translational initiation at the first uORF (uORF1) and reinitiation at the second uORF (uORF2) or third uORF (uORF3). Translational initiation and reinitiation at these uORFs also inhibit the production of ATF4 protein, because the last uORF overlaps with the coding region. However, a high level of phosphorylated eIF2α leads to slow formation of the translational initiation complex and scans through uORF2 or uORF3, resulting in reinitiation at the ORF encoding ATF4^15^,^16^. Phosphorylation of eIF2α is catalysed by at least four kinases: general control nonderepressible 2 (GCN2), protein kinase RNA-activated (PKR), PKR-like ER kinase (PERK), and heme-regulated inhibitor kinase (HRI)^17^. Each kinase responds to a distinct type of stress, as shown in Fig. 1. For this reason, the eIF2α-ATF4 signalling pathway is referred to as the integrated stress response (ISR)^18^. A high level of phosphorylated eIF2α leads to not only translational induction of ATF4, but also to global repression of protein synthesis^19^. On the other hand, dephosphorylation of eIF2α is regulated by protein phosphatase 1 regulatory subunits 15a and 15b^20^,^21^.

Many researchers have attempted to elucidate the functions of eIF2α and ATF4 at the cellular level, as described above, and they have suggested an association between the ISR and various human diseases^22^. However, the physiological and pathological roles of the ISR remain to be fully understood in mammals, because there is no suitable *in vivo* model for studying the response at the whole-body level. Here, we reported on our development of a transgenic mouse model for imaging ATF4-related cellular stress response. This mouse model is transduced with a transgene that contains an ATF4 uORF region and a luciferase-encoding region. Under conditions of stress that cause phosphorylation of eIF2α, luminescence signals are generated in tissues or cells collected from the transgenic mice, as well as in the whole bodies of the mice. Consequently, this mouse model is a useful tool for easily monitoring the ISR *in vivo*. In the future, by using this mouse model, we might be able to investigate the physiological and pathological roles of the ISR and to develop clinical drugs for treating ISR-associated diseases.

## Results

### Design and construction of UMAI reporter gene

**Figure 2:**
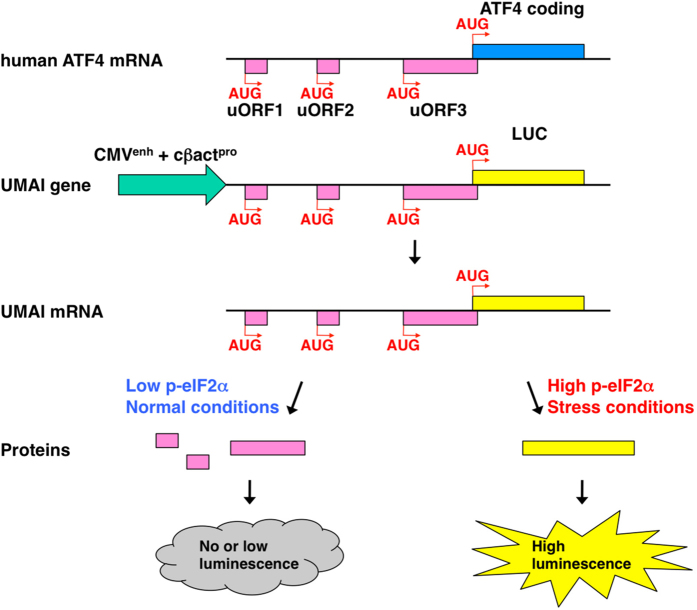
Schematic of the UMAI function. To detect the ISR, the UMAI gene was designed as follows; uORFs (pink)-containing human ATF4 gene whose coding region (blue) is replaced with that of luciferase (yellow) is regulated by a promoter/enhancer (green) activated constitutively. The mRNA transcribed from the UMAI gene should perform similarly to endogenous ATF4 mRNA during cellular stress. Thus, uORFs of the UMAI gene are dominantly translated under normal conditions, although luciferase is alternatively translated under stress conditions. We should therefore be able to monitor the ISR *in vivo* by measurement of luminescence signals from animals and cultured cells introduced with the UMAI gene.

**Figure 3:**
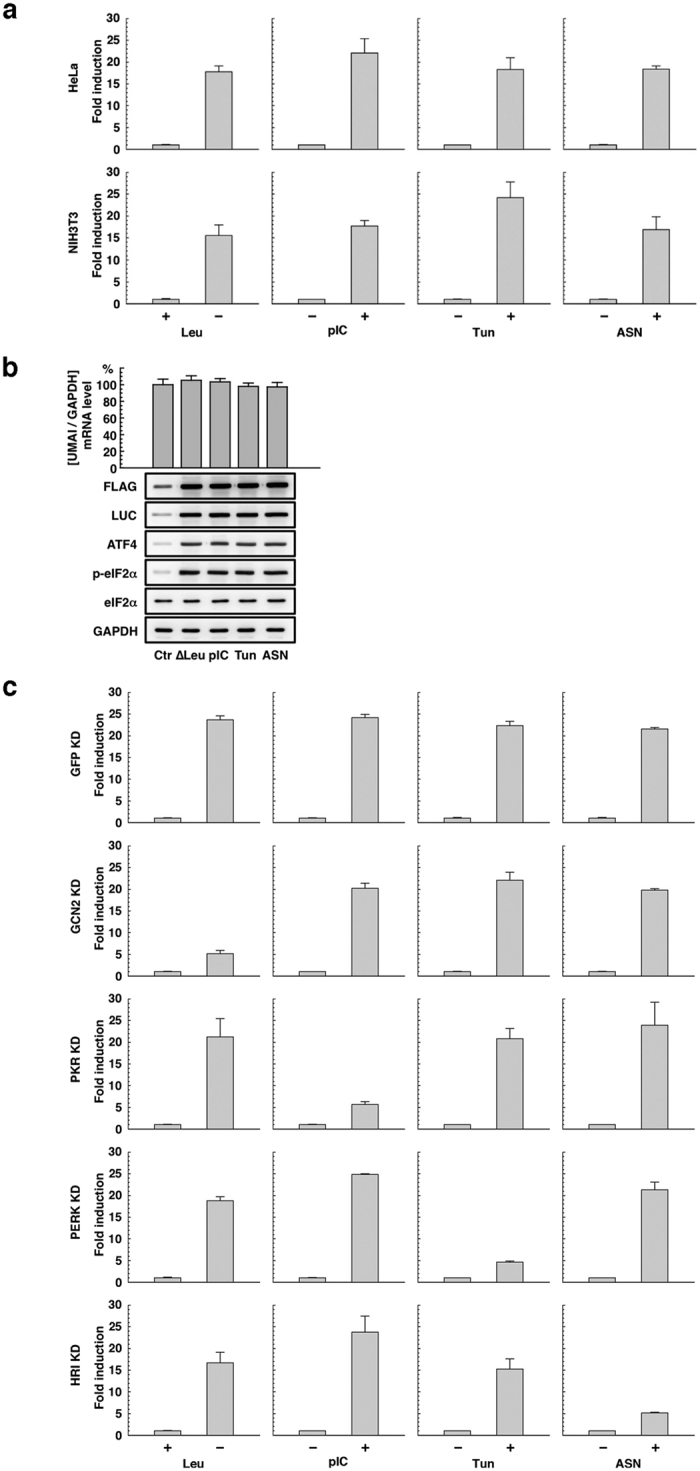
Characterization of UMAI ***in vitro***. (**a**) Luciferase activity in HeLa and NIH3T3 cells transfected with the UMAI plasmid and treated with various stressors to induce the ISR. (**b**, upper) Quantitative PCR analysis of UMAI mRNA expression in NIH3T3 cells transfected with the UMAI plasmid and treated with various stressors to induce the ISR by using a specific probe/primer set. The expression level of endogenous GAPDH mRNA was measured as an internal standard. This histogram is shown as mean (column) ± S.E.M (error bar) from triplicate experiments. (**b**, lower) Western blot analysis, performed by using an anti-FLAG antibody and an anti-luciferase antibody, of UMAI protein expression in NIH3T3 cells transfected with the UMAI plasmid and treated with the various stressors to induce the ISR: expression levels of total ATF4 protein, phosphorylated eIF2α protein, total eIF2α protein, and total GAPDH protein were examined as comparative and internal standards. (**c**) Luciferase activity in NIH3T3 cells transfected with the UMAI plasmid and the appropriate eIF2α kinase KD plasmid and treated with the various stressors to induce the ISR. Luciferase activity in NIH3T3 cells transfected with the UMAI plasmid and GFP KD plasmid was examined as a control experiment. These histograms are shown as mean (column) ± S.E.M (error bar) from triplicate experiments.

As described above, cellular stress activates specific eIF2α kinases, leading to translational induction of ATF4. To permit the detection of such ISRs, we designed a gene construct in which the coding region of ATF4 is replaced with that of luciferase; this construct is also activated transcriptionally by a constitutive promoter/enhancer and regulated translationally by uORFs. We named this gene construct ‘UMAI’, from uORFs mediated ATF4 indicator. The mRNA transcribed from the UMAI construct should behave similarly to endogenous ATF4 mRNA during cellular stress. Thus, uORFs of the UMAI construct are dominantly translated under normal conditions, whereas luciferase is alternatively translated under conditions of stress. We should therefore be able to monitor the ISR *in vivo* by measurement of luminescence signals from animals or culture cells introduced with the UMAI construct (Fig. 2). By developing the UMAI construct on the basis of human ATF4 gene and introducing the construct into HeLa or NIH3T3, we were able to confirm that luciferase activity increased markedly in response to deprivation of a specific amino acid (Leu) as well as to treatments with poly(I)poly(C) nucleotide (pIC), which mimics virus infection; tunicamycin (Tun), an endoplasmic reticulum (ER) stress inducer; and sodium arsenite (ASN), an oxidative stress inducer (Fig. 3a). Whereas mRNA transcribed from the UMAI construct was expressed at the same level under both normal and stress conditions, the indicator protein translated from the construct was expressed at a higher level under stress conditions than under normal conditions, in a similar manner to endogenous ATF4 protein and phosphorylated eIF2α protein (Fig. 3b).

The uORFs of the ATF4 mRNA are conserved among vertebrates^15^,^16^. Consequently, the UMAI can also be constructed on the basis of the mouse ATF4 gene (Fig. S1). As a trial, we also constructed the mouse-derived UMAI, and we compared the stress responsivity in NIH3T3 of the human and mouse indicators. The human-type UMAI showed a slightly higher responsivity than the mouse type did, although both indicators were robustly activated under all the stress conditions that we examined (Fig. S2). This result persuaded us to generate transgenic mice by using the human type of UMAI construct.

By introducing the human type of UMAI construct into NIH3T3 cells that were impaired in a specific eIF2α kinase, we confirmed that responsivity of the UMAI to stress is dependent on each eIF2α kinase as follows. Knockdown (KD) of GCN2, PKR, PERK, and HRI obviously diminished the responsivity of the UMAI to Leu (−), pIC (+), Tun (+), and ASN (+), respectively (Fig. 3c and Fig. S3). These results indicated that the UMAI construct is a functional tool for easily monitoring the ISR.

### Generation and characterization of UMAI transgenic mice

**Figure 4:**
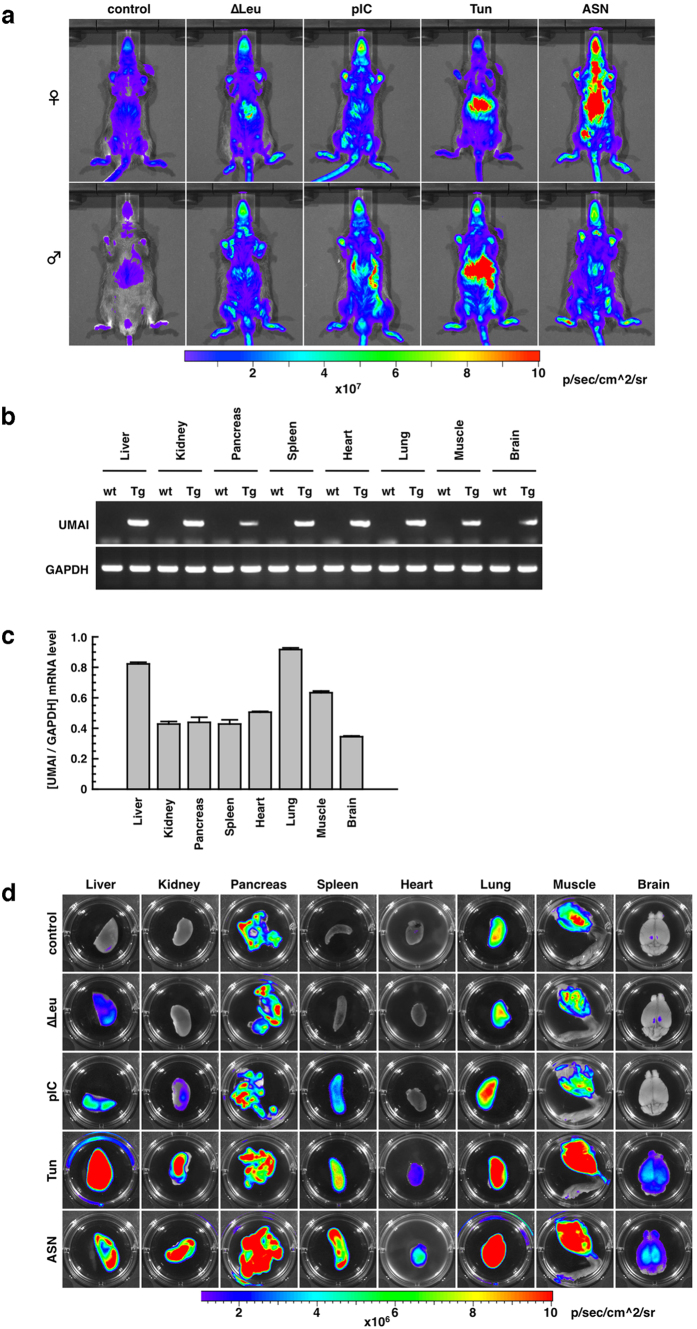
Characterization of UMAI *in vivo.* (**a**) Luminescence signals obtained from UMAI mice (12-week-old) at the F2 generation by *in vivo* imaging analysis at the whole-body level. (**b**) RT-PCR analysis of UMAI transgene expression in various tissues. GAPDH was used as an internal standard. Wild type, wt; transgenic, Tg. The tested RNA was extracted from UMAI mice (12-week-old, female) at the F2 generation. (**c**) Quantitative PCR analysis of the transgene expression in various tissues derived from UMAI mice. The tested RNA was extracted from UMAI mice (12-week-old, female) at the F2, F3, and F6 generations. This histogram is shown as mean (column) ± S.E.M (error bar) from 3 samples (F2, F3, and F6). GAPDH was used as an internal standard. (**d**) Luminescence signals obtained from various tissues of UMAI mice (12-week-old, female) at the F2 generation by *ex vivo* imaging analysis. The tissues for *ex vivo* imaging analysis were collected from UMAI mice treated with the various stressors to induce the ISR.

By microinjection of the UMAI construct into approximately 400 fertilized mouse eggs, we generated 27 UMAI transgenic mice (F0). Seven of these F0 mice had transgenic-positive offspring, but the other line did not. Offspring derived from the seven F0 mice were maintained as individual lines and analysed for *in vivo* imaging of the ISR. Six lines of these unexpectedly showed stronger luminescence signals under normal condition or weaker luminescence signals under stress conditions. One line, however, showed lower luminescence signals under normal conditions and higher luminescence signals under stress conditions (Fig. 4a). Expression of the UMAI mRNA in this mouse line was evaluated by RT-PCR analysis and by quantitative PCR analysis; these showed that the UMAI mRNA was expressed in all the examined organs, even under normal conditions (Fig. 4b), and that the expression level of the UMAI mRNA remained within a factor of three among these organs (Fig. 4c). From these results, we consider that this UMAI mouse line is a potential animal resource for investigating the ISR *in vivo. Ex vivo* analysis of the transgenic line also revealed significant UMAI activity under normal conditions, and stress-dependent differences in UMAI activity. In particular, under normal conditions, the pancreas, muscle, and lung collected from UMAI transgenic mice showed bright signals compared with the other organs. A Leu (−) diet modestly increased the UMAI activity of the liver, but had little effect on other organs. Administration of pIC also had moderately increased the UMAI activity of the liver, kidney, spleen, and lung, but had little effect on the pancreas, heart, muscle, or brain. On treatment with Tun and ASN, UMAI activity was robustly displayed in all the examined organs (Fig. 4d). These features of UMAI activity *in vivo* and *ex vivo* were also sustained in other generations (Figs S4 and S5), and were well correlated with endogenous ATF4 protein level and endogenous eIF2α phosphorylation level (Fig. S6).

Some F0 mice of unestablished lines were introduced with multiple copies of the transgene and other F0 mice were introduced with the transgene(s) in an unexpected manner. However, the F0 mouse of the line established as the UMAI mouse was introduced with a single copy of the transgene in the expected manner (Fig. S7a). The copy number of the transgene was sustained in subsequent generations of the established UMAI line (Fig. S7b). In addition CpG methylation was examined in the CMV enhancer region of the transgene introduced into the established UMAI mice. The CMV enhancer region was found to have low methylation level in various tissues of both young (10-week-old) and old (42-week-old) UMAI mice (Supplementary Table). The transgene was expressed with the same pattern in both male and female UMAI mice, and in both young and old UMAI mice (Fig. S8).

**Figure 5:**
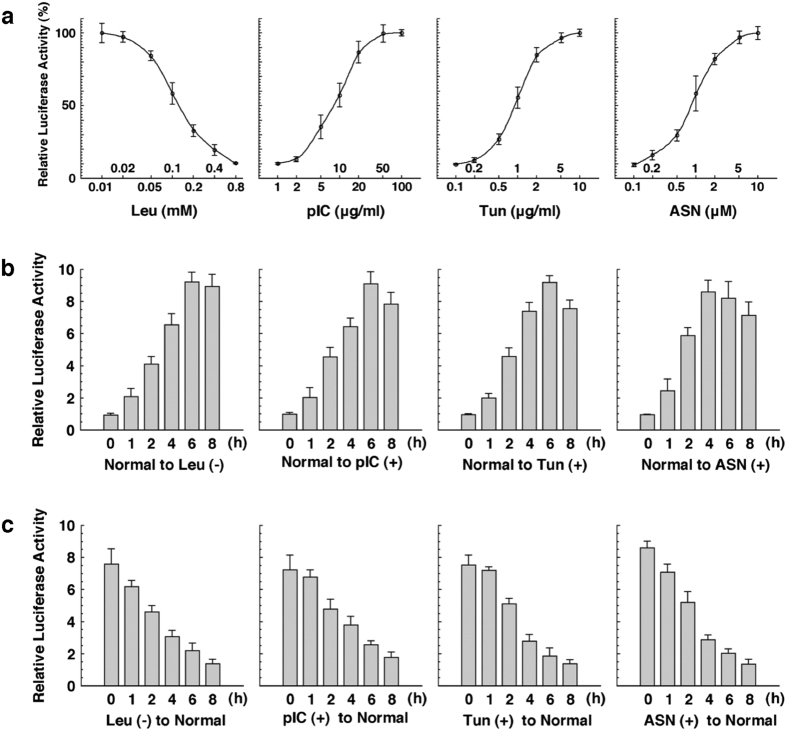
Evaluation of the ISR by using UMAI MEFs. (**a**) Luciferase activity in UMAI MEFs treated with the indicated concentration of leucine (Leu), poly(I)poly(C) nucleotide (pIC), tunicamycin (Tun), or sodium arsenite (ASN) for 6 h. (**b**) Luciferase activity in UMAI MEFs treated with Leu (−), pIC, Tun, and ASN for the indicated time. (**c**) Luciferase activity in UMAI MEFs treated with Leu (−), pIC, Tun, and ASN for 6 h and then cultured in a normal medium for the indicated time. Each graph is shown as mean (plot or column) ± S.E.M (error bar) from triplicate experiments.

By using mouse embryonic fibroblasts (MEFs) derived from this UMAI line, we were able to obtain data on how UMAI activity varied with the intensity and duration of stress treatment. UMAI activity was found to change widely in the ranges of the following stress intensity: Leu (0.01–0.8 mM), pIC (1–100 μg/mL), Tun (0.1–10 μg/mL), and ASN (0.1–10 μM) (Fig. 5a). Under all conditions [Leu (−), pIC (+), Tun (+), and ASN (+)], the UMAI activity reached a peak 4–6 hours after the initiation of stress treatment (Fig. 5b), and reverted to the basal line 8 hours after removal of the stress (Fig. 5c). These UMAI activities were well correlated with both the expression level of endogenous ATF4 protein and the phosphorylation level of endogenous eIF2α (Figs S9 and S10).

## Discussion

It has been reported that the ISR is associated with various diseases, and this association needs to be investigated by using sophisticated mouse models. Here, we developed UMAI constructs and UMAI mice. Under conditions stimulating eIF2α-ATF4 pathway, cultured cell lines introduced with UMAI construct expectedly induce translation of reporter protein and show high reporter activity (Fig. 3a,b). The UMAI mice also constitutively expressed the transgene-derived mRNA in all the examined organs and they showed the expected increase in reporter activity under conditions stimulating eIF2α-ATF4 pathway (Fig. 4). These features indicate that the UMAI construct and UMAI mice are useful biological resources for the investigation of the ISR *in vitro* and *in vivo*. In addition, UMAI activity is finely regulated in a stress-intensity-dependent manner and a stress-duration-dependent manner (Fig. 5), and it is profoundly influenced by the function of eIF2α kinases (Fig. 3c). These findings suggest that UMAI might contribute to research on the roles of GCN2, PKR, PERK, and HRI in detail. The performance demonstrated by our construct and the model mouse is reasonable, because UMAI-like constructs have previously been reported to function as indicators for the ISR^23^. The specificity of the response of the UMAI to the various stresses shown in our KD experiments is coincided with that of previous reports on the disruption of eIF2α kinases^15^,^24^,^25^,^26^. We therefore believe that UMAI is a reliable experimental tool.

A variety of model mice with transgene constructs consisting of transcriptional regulatory elements and a luminescent/fluorescent reporter have been developed for imaging of biological response *in vivo*. Among these, the CARE-LUC mouse model is designed to express luciferase by transcriptional regulation with ATF4 and its binding elements, and this model is used for the evaluation of eIF2α-ATF4 signalling under stress conditions that activate GCN2 or PERK^27^. However, there is concern that the signals detected from CARE-LUC mice might reflect factors other than the ISR. This is because the expression level of ATF4 is regulated not only by ISR-related translation, but also by promoter-related transcription and ubiquitin/proteasome system-related protein degradation, as described in the introduction, and a variation in the expression level of ATF4 has a transcriptional effect on the CARE-LUC gene. On the other hand, the reporter expression of UMAI is regulated by translational control of uORFs, and not by the transcriptional activating function of ATF4. Consequently, UMAI should be used in monitoring the ISR *in vivo* to avoid the effects of the expression level of endogenous ATF4.

UMAI mice show a high luciferase activity in the pancreas, muscle, and lung, even under normal conditions. We do not have a clear answer to the question of what stress or which eIF2α kinase gives rise to the signal from those tissues. However, we can obtain some hints from other model mice previously developed for imaging of cellular stress. OKD48 mice have a reporter gene regulated by Keap1 and Nrf2, and they have been developed for imaging oxidative stress *in vivo*^28^,^29^. These mice show hardly reporter signal in any tissue under normal conditions. ERAI mice have a reporter gene that is regulated by IRE1α and XBP1, and they have been developed for imaging ER stress *in vivo*^30^,^31^. These mice show a high reporter activity in the pancreas and the muscle, even under normal conditions. The data from other mouse model suggest that the signals in the pancreas and the muscle tissue of UMAI mice might be caused by PERK activated by the physiological ER stress in these tissues.

UMAI mice have a unique reporter gene translationally controlled by uORFs of ATF4 and they are useful in low-invasiveness monitoring of eIF2α-ATF4 signalling under stress conditions that activate GCN2, PKR, PERK, and HRI. Consequently, by using UMAI mice, we might be able to address various problems associated with the ISR in diseases and in pharmaceutical development. For example, by mating UMAI mice with a disease mouse model, we might be able to examine temporal change in the ISR and ATF4 translational activation with the progression of the disease in identical mice. By using UMAI mice treated with a certain drug, we might easily examine the effects of that drug on the ISR and ATF4 translational activation at the whole-body level of mice. Thus, UMAI mice might be useful in developing new diagnostic and therapeutic methods for ATF4-related diseases. We therefore expect that UMAI mice will form a valuable resource for medical science.

## Methods

### Cell culture, transfection, and treatment

MEFs were collected in accordance with a previously described procedure^32^. NIH3T3 cells, HeLa cells, and MEFs were cultured in Dulbecco’s modified Eagle’s medium supplemented with 10% fetal bovine serum at 37 °C under 5% CO_2_. The calcium phosphate–DNA precipitation method was used to introduce plasmids into NIH3T3 cells and HeLa cells. To induce an amino acid-deprivation response, cells were treated in the absence or presence of the indicated concentration of l-leucine for 6 h or the indicated time. To induce an antivirus response, cells were treated with 100 μg/mL or the indicated concentration of poly(I)poly(C) nucleotide (#27-4732-01; GE Healthcare Life Sciences, Chicago, IL) for 6 h or the indicated time. To induce an ER stress response, cells were treated with 10 μg/mL or the indicated concentration of tunicamycin (#T7765; Sigma-Aldrich, Saint Louis, MO) for 6 h or the indicated time. To induce an oxidative stress response, cells were treated with 10 μM or the indicated concentration of sodium arsenite (#106277; Merck Millipore, Billerica, MA) for 6 h or the indicated time. Leucine (−) medium was obtained commercially (#1642149; MP Biomedicals, Santa Ana, CA).

### Gene constructs

pCAX-LUC-F(XhoI) was made by insertion of a LUC-F fragment into the HindIII/BamHI sites of pCAX. The LUC-F fragment encoding luciferase with Flag-tag on its 3′ terminal was produced by PCR using 5′-ccc aag ctt cca cca tgc tcg agg aag acg cca aaa aca taa ag-3′ as the forward primer, 5′-cgc gga tcc tta ctt gtc atc gtc gtc ctt gta gtc cac ggc gat ctt tcc gcc ctt c-3′ as the reverse primer, and pGL3-basic (#E1751; Promega, Fitchburg, WI) as the template.

The pCAX-hATF4(uORFs)-LUC-F, human type of UMAI construct was made by insertion of an hATF4(uORFs) fragment into the HindIII/XhoI sites of pCAX-LUC-F(XhoI). The hATF4(uORFs) fragment was produced by PCR using 5′-ccc aag ctt gtt ttc tac ttt gcc cgc cca cag atg tag ttt tct ctg c-3′ as the forward primer, 5′-ccg ctc gag cat ggt gca gtg ctt tgc tgg aat caa cg-3′ as the reverse primer, and human ATF4 cDNA as the template.

The pCAX-mATF4(uORFs)-LUC-F, mouse type of UMAI construct was made by insertion of mATF4(uORFs) fragment into the HindIII/XhoI sites of pCAX-LUC-F(XhoI). The mATF4(uORFs) fragment was produced by PCR by using 5′-ccc aag ctt ttt tct gct tgc tgt ctg ccg gtt tga gtt gtg c-3′ as the forward primer, 5′-ccg ctc gag cat gtt gtg ggg ctt tgc tgg att cca gg-3′ as the reverse primer, and mouse ATF4 cDNA as the template.

To produce pSUPER-mGCN2, pSUPER-mPKR, pSUPER-mPERK, and pSUPER-mHRI for KD of the genes coding the corresponding eIF2α kinases, gta tac gtg caa gtg gaa c, ctt agt act tcg gga cct c, tca tca gca ctt tag atg g, and cac ttg agc cat gtg cac g, respectively, were selected as target sequences and cloned into pSUPER (#VEC-PBS-0002; Oligoengine, Seattle, WA) in accordance with supplier’s instructions. As a control experiment, pSUPER-GFP was produced in the same manner with gca agc tga ccc tga agt t as the target sequence.

### Luciferase reporter assay

NIH3T3 cells and HeLa cells were seeded in 12-well plates at 5 × 10^4^ cells/well, then transfected with plasmid DNA (2 μg/well). 30 h after transfection and 6 h or the indicated time after the appropriate stress treatment, the cells were lysed for luciferase assay. phRL-TK (#E6241; Promega) was used as an internal control in this assay. UMAI MEFs were also seeded in 12-well plates at 5 × 10^4^ cells/well, treated with the indicated stress, and then lysed for luciferase assay. Luciferase activity was measured by using a luciferase assay system (#E1910; Promega) and a luminometer (#LB960; Berthold Technologies, Bad Wildbad, Germany).

### Transgenic mice and stress treatment

The 4.5-kb SpeI-PvuII fragment of pCAX-hATF4(uORFs)-LUC-F was microinjected as a transgene into fertilized mouse eggs (C57BL/6), and the transgenic offspring were screened by PCR using the following primers: 5′-ggc cac cat ggc gta tta gg-3′ and 5′-tct tcc agc gga tag aat gg-3′. To induce an amino acid-deprivation response, mice were fed with a leucine-free diet (#A08051503; Research Diets, New Brunswick, NJ) for 24 hours. An amino acid-complete diet (#A08051501; Research Diets) was given to mice as a control. To induce an antivirus response, mice were injected intraperitoneally with poly(I)poly(C) nucleotide (5 μg per gram body weight) 6 h before imaging analysis. To induce an ER stress response, mice were injected intraperitoneally with tunicamycin (500 ng per gram body weight) 6 h before imaging analysis. To induce an oxidative stress response, mice were injected intraperitoneally with sodium arsenite (15 μg per gram body weight) 6 h before imaging analysis. Poly(I)poly(C) nucleotide (#27-4732-01; GE Healthcare Life Sciences), tunicamycin (#T7765; Sigma-Aldrich), and sodium arsenite (#106277; Merck Millipore) were obtained commercially. Heterozygous UMAI mice were used in all the experiments of this research. The experimental protocols that involved animals were approved by the Animal Studies Committees at Kumamoto University, TransGenic Inc., and Kanazawa Medical University (A25-051, 2016J03, and 2016-107, respectively); all experiments were performed in accordance with the appropriate institutional guidelines.

### RT-PCR and quantitative PCR

Total RNA derived from plasmid-transfected culture cells and various tissues of wild-type and UMAI mice was prepared by using Isogen reagent (#311-02501; Nippon Gene, Tokyo, Japan). The cDNA was synthesized by using the SuperScript first-strand synthesis system (#11904-018; Invitrogen, Waltham, MA) in accordance with the manufacturer’s instructions. UMAI cDNA was amplified by 35 cycles of PCR with 5′-ggc cac cat ggc gta tta gg-3′ and 5′-act cag cgt aag tga tgt cc-3′ as primers. GAPDH cDNA was also amplified by 35 cycles of PCR with 5′-ctg aac ggg aag ctc act gg-3′ and 5′-cac cac cct gtt gct gta gc-3′ as primers. Quantitative PCR analysis of each transcript was performed by using TaqMan probe and StepOnePlus (#4376592; Applied Biosystems, Waltham, MA), in accordance with the manufacturer’s instructions. Probe/primer sets: Mm00469222_m1, Mm01235643_m1, Mm00438700_m1, Mm01202300_m1, and 4352339E (Applied Biosystems) were used for quantification of GCN2, PKR, PERK, HRI, and GAPDH transcripts, respectively. UMAI transcripts were quantified by using 5′-tgc aca tat cga ggt gga cat c-3′ as the forward primer, 5′-tgc caa ccg aac gga cat-3′ as the reverse primer, and 5′-FAM-ctt acg ctg agt act tcg-MGB-3′ as the probe.

### Western blot analysis

The cells were lysed in SDS sample buffer (50 mM Tris-HCl, pH 6.8, 2% SDS, 50 mM DTT, 10% glycerol, and 1 mg/mL bromophenol blue). The lysate was heated to 98 °C for 10 min, and SDS-PAGE was performed to separate the proteins in the lysate. After electrophoresis, the proteins were electrotransferred onto a PVDF microporous membrane and immunodetected with the appropriate antibody. The following antibodies were used for Western blot analysis: anti-FLAG polyclonal antibody (#F7425; Sigma-Aldrich), anti-luciferase polyclonal antibody (#G7541; Promega), anti-ATF4 monoclonal antibody (#11815; Cell Signaling Technology, Beverly, MA), anti-eIF2α monoclonal antibody (#5324; Cell Signaling Technology), anti-phospho-eIF2α (Ser51) monoclonal antibody (#3398; Cell Signaling Technology), and anti-GAPDH monoclonal antibody (#2118; Cell Signaling Technology).

### Imaging of luminescence signals *in vivo* and *ex vivo*

UMAI mice were injected intraperitoneally with d-luciferin (150 mg/g body weight) in PBS 10 min before imaging analysis. Tissues were collected surgically from the UMAI mice 10 min after luciferin injection and were immersed in a 300 mg/mL solution of d-luciferin. Imaging analyses were performed by using IVIS (#Lumina; Perkin Elmer, Waltham, MA). After a grey-scale photograph had been acquired, luminescence images were obtained under the following conditions. For *in vivo* analysis: exposure, 60 s; field of view, 12.5 cm; binning (resolution) factor 2; open filter. For *ex vivo* analysis: exposure, 60 s; field of view, 5 cm, binning (resolution) factor 2; open filter.

## Additional Information

**How to cite this article**: Iwawaki, T. *et al*. Transgenic mouse model for imaging of ATF4 translational activation-related cellular stress responses *in vivo. Sci. Rep.* **7**, 46230; doi: 10.1038/srep46230 (2017).

**Publisher's note:** Springer Nature remains neutral with regard to jurisdictional claims in published maps and institutional affiliations.

## Supplementary Material

Supplementary Information

## Figures and Tables

**Figure 1:**
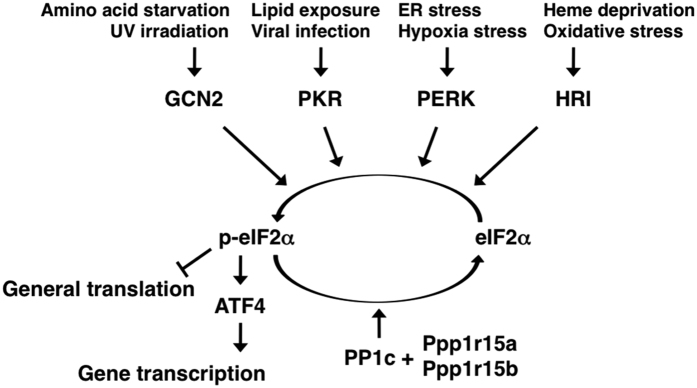
Schematic of the integrated stress response (ISR). Four eIF2α kinases (GCN2, PKR, PERK, and/or HRI) are specifically activated under certain stress conditions. A high level of phosphorylated eIF2α leads to global repression of protein synthesis and to translational induction of ATF4. These responses alleviate the burden of the stress factor and activate genes for stress resistance. On the other hand, dephosphorylation of eIF2α is regulated by Ppp1r15a and/or Ppp1r15b.
