# Supplementary material for: Transgenic mouse model for imaging of ATF4 translational activation-related cellular stress responses in vivo
Source: Sci Rep. 2017 Apr 7;7:46230. doi: 10.1038/srep46230 (PMC5384252; doi:10.1038/srep46230)
Supplement: Supplementary Information [file srep46230-s1.pdf]

Supplementary information for

Transgenic mouse model for imaging of ATF4 translational activation-related cellular stress responses *in vivo*

Takao Iwawaki, Ryoko Akai, Takae Toyoshima, Naoki Takeda, Tomo-o Ishikawa, and Ken-ichi Yamamura

Contents

Supplementary Methods
Supplementary Figure 1 - 10
Supplementary Table

SUPPLEMENTARY METHODS

ELISA

Protein was extracted from mouse tissues and mouse embryonic fibroblasts (MEFs) by using Mammal Tissue Extraction Reagent (#AR0101; Boster Biological Technology, Pleasanton, CA). Signals were detected for the reactions of coated antigens with anti-ATF4 monoclonal antibody (#11815; Cell Signaling Technology, Beverly, MA), anti-eIF2α monoclonal antibody (#5324; Cell Signaling Technology), anti-phospho-eIF2α (Ser51) monoclonal antibody (#3398; Cell Signaling Technology), and anti-GAPDH monoclonal antibody (#2118; Cell Signaling Technology), in accordance with the manufacturer's protocol (https://www.ptglab.com/media/1604/protocols-for-web_indirect-elisa.pdf).

Southern blot analysis

Genome DNA was extracted from mouse tail tips in accordance with the standard procedure. Aliquots (10 μg) of genome DNA were digested with HindIII/BamHI, loaded into separate lanes of 0.8% agarose gels, and transferred onto nylon membranes (#60207; PALL, Port Washington, NY). Hybridization was performed in H solution (0.5 M Na_2HPO_4, 1 mM EDTA, and 7% SDS) at 65 °C for 16 h. The membranes were then washed four times with W solution (40 mM Na_2HPO_4 and 1% SDS) at 65 °C for 10 min. The ^{32}P-labelled luciferase-coding region derived from pGL3-basic (#E1751; Promega, Fitchburg, WI) was used as a probe to detect the UMAI transgene.

Quantitative PCR analysis for copy-number analysis of the transgene in UMAI mice

Quantitative PCR analysis of genome DNA was performed by using the TaqMan probe and StepOnePlus (#4376592; Applied Biosystems, Waltham, MA), in accordance with the manufacturer's instructions. The UMAI transgene was quantified by using 5'-tgc aca tat cga ggt gga cat c-3' as the forward primer, 5'-tgc caa ccg aac gga cat-3' as the reverse primer, and 5'-FAM-ctt acg ctg agt act tcg-MGB-3' as the probe. Probe/primer sets Mm00607939_s1, (Applied Biosystems) were used for quantification of β-actin.

Methylation analysis of CMV enhancer in transgenes of UMAI mice

The genome DNA was extracted from various mouse tissues in accordance with a standard procedure. Bisulphite modification of the extracted DNA was performed by using a commercial research kit (#ME002; Genetic Signatures, Darlinghurst, Australia). The CMV enhancer region was amplified by PCR using 5'-ccc aag ctt tag tta tta ata gta at-3' as the forward primer, 5'-cgc gga tcc cac aaa ata att aaa aaa taa ata aat ac-3' as the reverse primer, and the modified DNA as the template. The PCR product was inserted into the HindIII/BamHI sites of pBluescript II SK(–) (#212206; Agilent Technologies, Santa Clara, CA), and sequenced by using universal M13-47 primer. The sequence data were analysed with an online tool (http://quma.cdb.riken.jp/).

SUPPLEMENTARY FIGURE LEGENDS

Supplementary Figure 1

Schematic of the UMAI using the mouse ATF4 gene

The uORFs (pink)-containing the mouse ATF4 gene whose coding region (blue) is replaced with that of luciferase (yellow) is regulated by promoter/enhancer (green) activated constitutively. The UMAI using the mouse ATF4 gene should show a similar performance to that using the human one.

Supplementary Figure 2

Comparison of the stress responses of human-derived UMAI and mouse-derived UMAI

The histograms show the luciferase activity in NIH3T3 cells transfected with the appropriate UMAI plasmid and treated with the various stressors to induce the ISR, and they are shown as mean (column) ± S.E.M (error bar) from triplicate experiments.

Supplementary Figure 3

Confirmation of KD efficiency

Expression levels of GCN2, PKR, PERK, and HRI mRNA in NIH3T3 cells transfected with the various KD plasmids were measured by quantitative PCR analysis. GFP KD was used as a negative control. The expression level of endogenous GAPDH mRNA was used as an internal standard. This histogram is shown as mean (column) ± S.E.M (error bar) from triplicate experiments.

Supplementary Figure 4

Luminescence signals obtained from UMAI mice at the whole-body level

(a) *in vivo* imaging analysis of UMAI mice (12-week-old) at the F3 generation. (b) *in vivo* imaging analysis of UMAI mice (12-week-old) at the F6 generation.

Supplementary Figure 5

Luminescence signals obtained from UMAI mice at the collected-tissue level

(a) *ex vivo* imaging analysis of UMAI mice (12-week-old, female) at the F3 generation. (b) *ex vivo* imaging analysis of UMAI mice (12-week-old, female) at the F6 generation. The tissues for *ex vivo* imaging analysis were collected from UMAI mice treated with the various stressors to induce the ISR.

Supplementary Figure 6

Expression level of endogenous ATF4 and phosphorylation level of endogenous eIF2α in various tissues derived from UMAI mice

The upper histogram shows the expression level of endogenous ATF4 protein. The expression level of endogenous GAPDH protein was used as an internal standard. The lower histogram shows the phosphorylation level of endogenous eIF2α protein. The total level of endogenous eIF2α protein was used as an internal standard. The tested protein was extracted from UMAI mice (12-week-old, female) at the F2, F3, and F6 generations. These histograms are shown as mean (column) ± S.E.M (error bar) from three samples (F2, F3, and F6).

Supplementary Figure 7

Copy number of the transgene in UMAI mice

(a) Southern blot analysis of the transgene in UMAI mice (F0 generation). The upper panel shows an autoradiography of the UMAI gene. The lower panel shows the results of ethidium bromide staining of the genome DNA loaded onto agarose gel. (b) Quantitative PCR analyses of the transgene were performed by using genome DNA derived from UMAI mice at the F2 and F6 generations (*n* = 3). β-actin was used for normalization.

Supplementary Figure 8

Comparison of UMAI expression in male and female mice and in young and old mice

Quantitative PCR analyses of the transgene were performed in various tissues derived from UMAI mice. These histograms are shown as mean (column) ± S.E.M (error bar) from three mice (*n* = 3). GAPDH was used as an internal standard.

Supplementary Figure 9

Expression level of endogenous ATF4 in UMAI MEFs

(a) Expression levels of endogenous ATF4 in UMAI MEFs treated with the indicated concentration of leucine (Leu), poly(I)poly(C) nucleotide (pIC), tunicamycin (Tun), or sodium arsenite (ASN) for 6 h. (b) Expression level of endogenous ATF4 in UMAI MEFs treated with Leu (–), pIC, Tun, and ASN for the indicated time. (c) Expression level of endogenous ATF4 in UMAI MEFs treated with Leu (–), pIC, Tun, and ASN for 6 h and then

cultured in a normal medium for the indicated time. Each graph is shown as mean (plot or column) ± S.E.M (error bar) from triplicate experiments.

Supplementary Figure 10

Phosphorylation level of endogenous eIF2α in UMAI MEFs

(a) Phosphorylation level of endogenous eIF2α in UMAI MEFs treated with the indicated concentration of leucine (Leu), poly(I)poly(C) nucleotide (pIC), tunicamycin (Tun), or sodium arsenite (ASN) for 6 h. (b) Phosphorylation level of endogenous eIF2α in UMAI MEFs treated with Leu (–), pIC, Tun, and ASN for the indicated time. (c) Phosphorylation level of endogenous eIF2α in UMAI MEFs treated with Leu (–), pIC, Tun, and ASN for 6 h and then cultured in a normal medium for the indicated time. Each graph is shown as mean (plot or column) ± S.E.M (error bar) from triplicate experiments.

Supplementary Table

Methylation analysis of CMV enhancer in the transgene of UMAI mice

The tested genome DNA was extracted from various tissues of male and female UMAI mice. The methylation analysis was performed for 16 samples (8 male and 8 female) from each tissue and each age.

Supplementary Figure 1

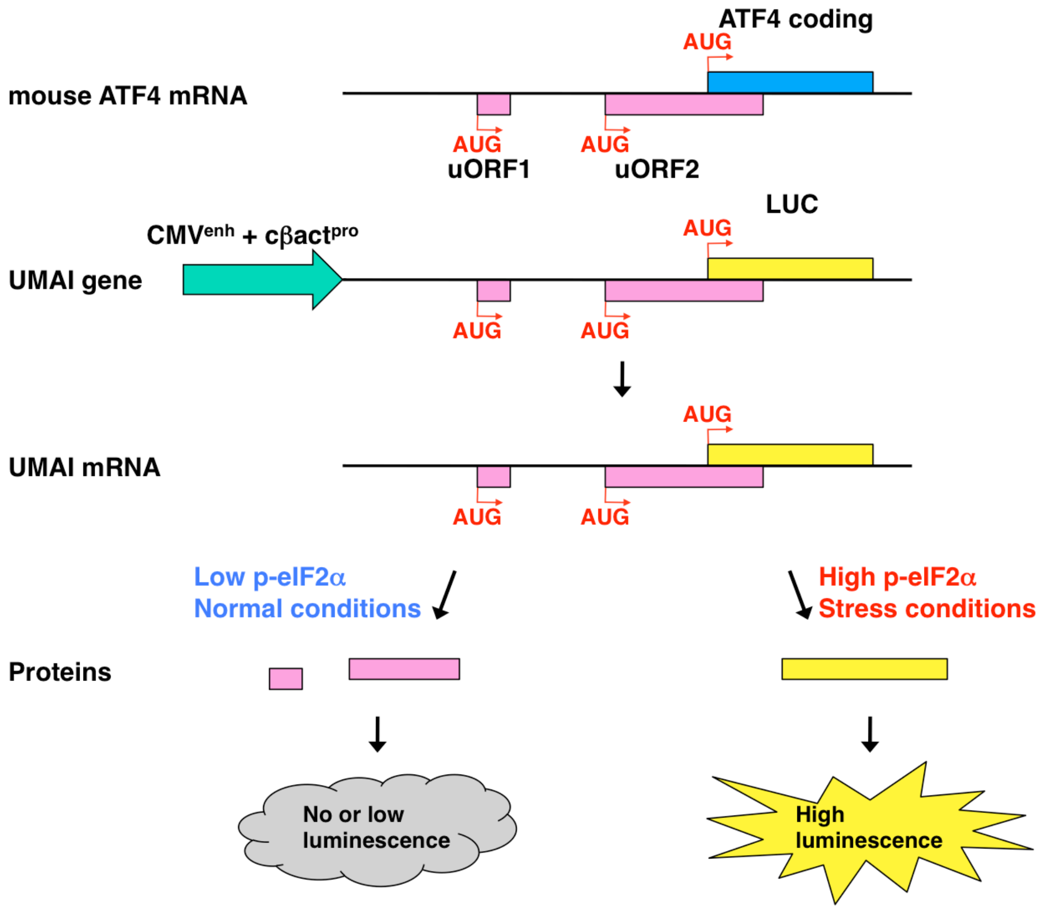

Supplementary Figure 2

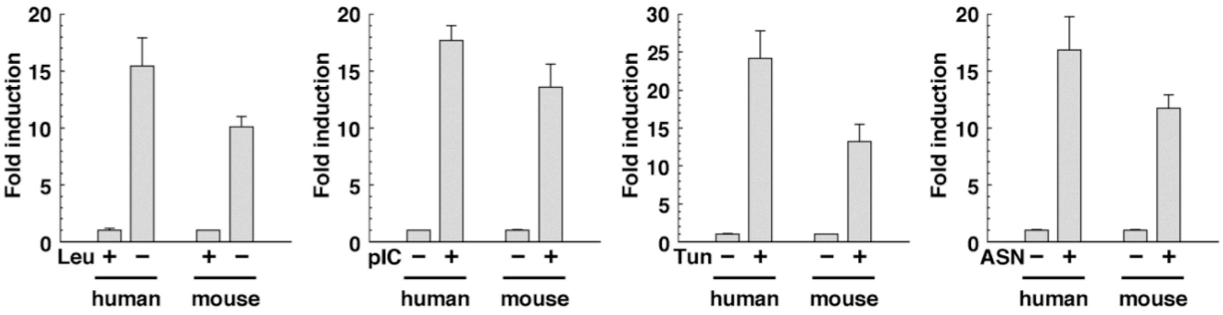

Supplementary Figure 3

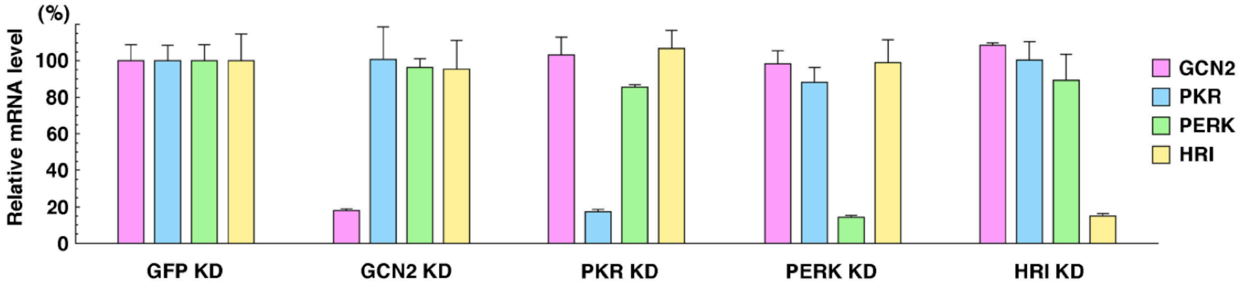

Supplementary Figure 4

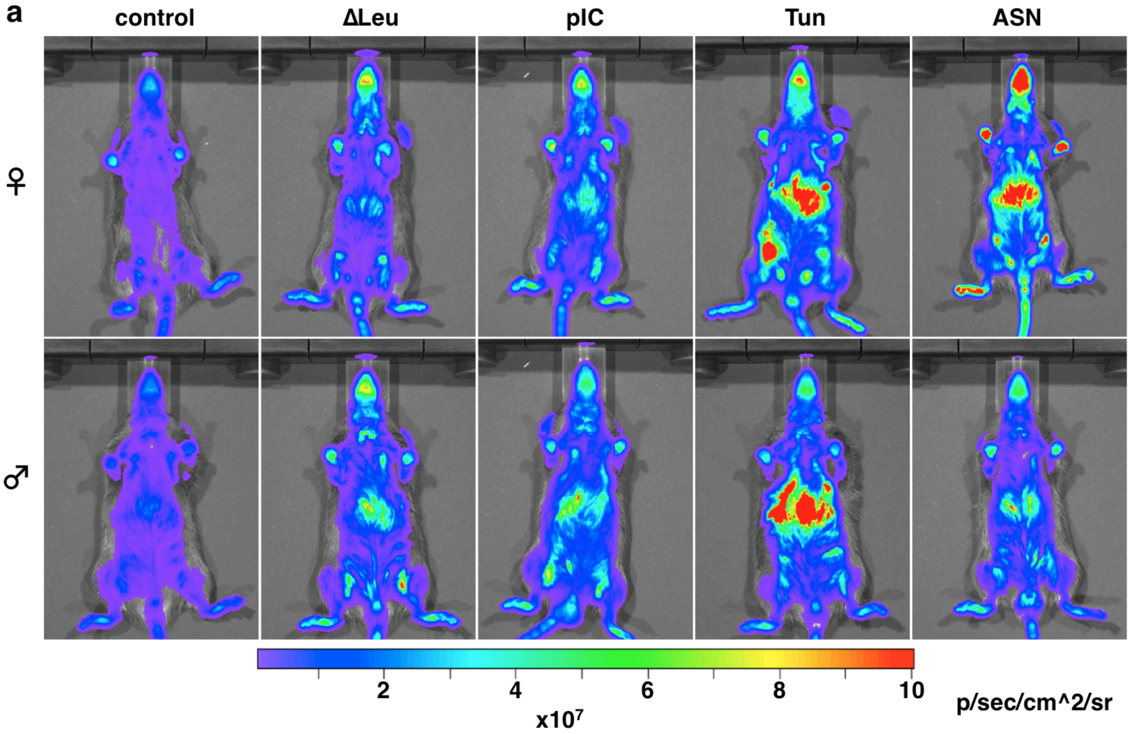

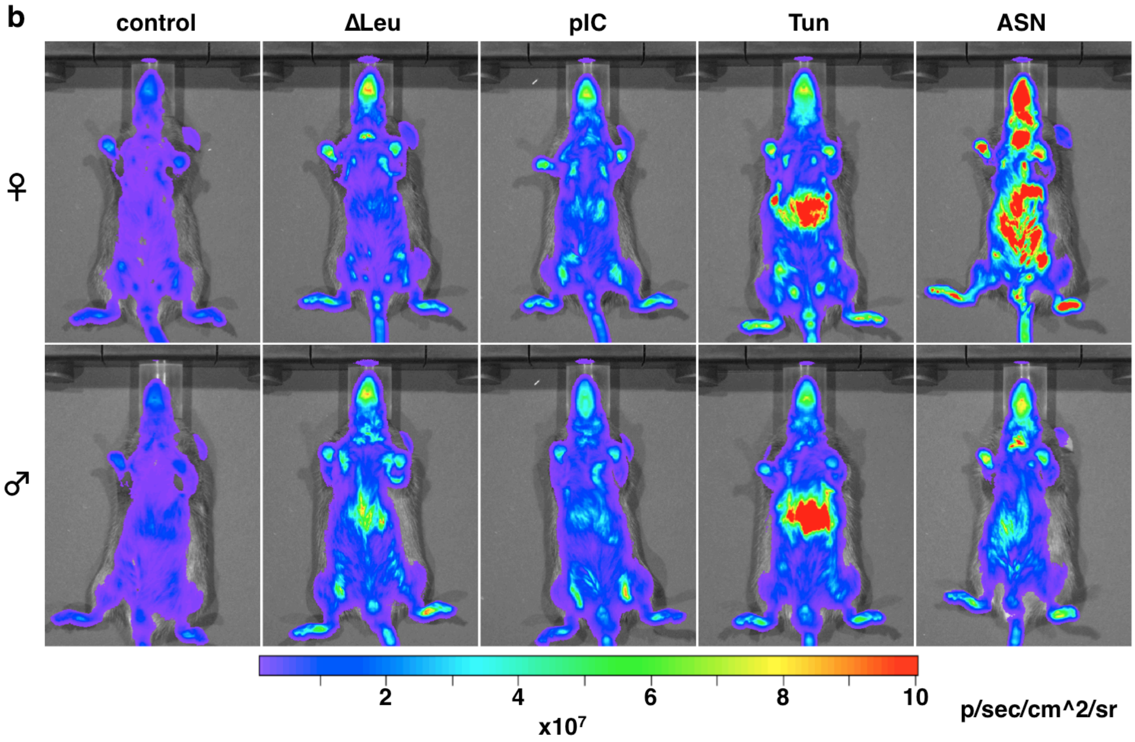

Supplementary Figure 5

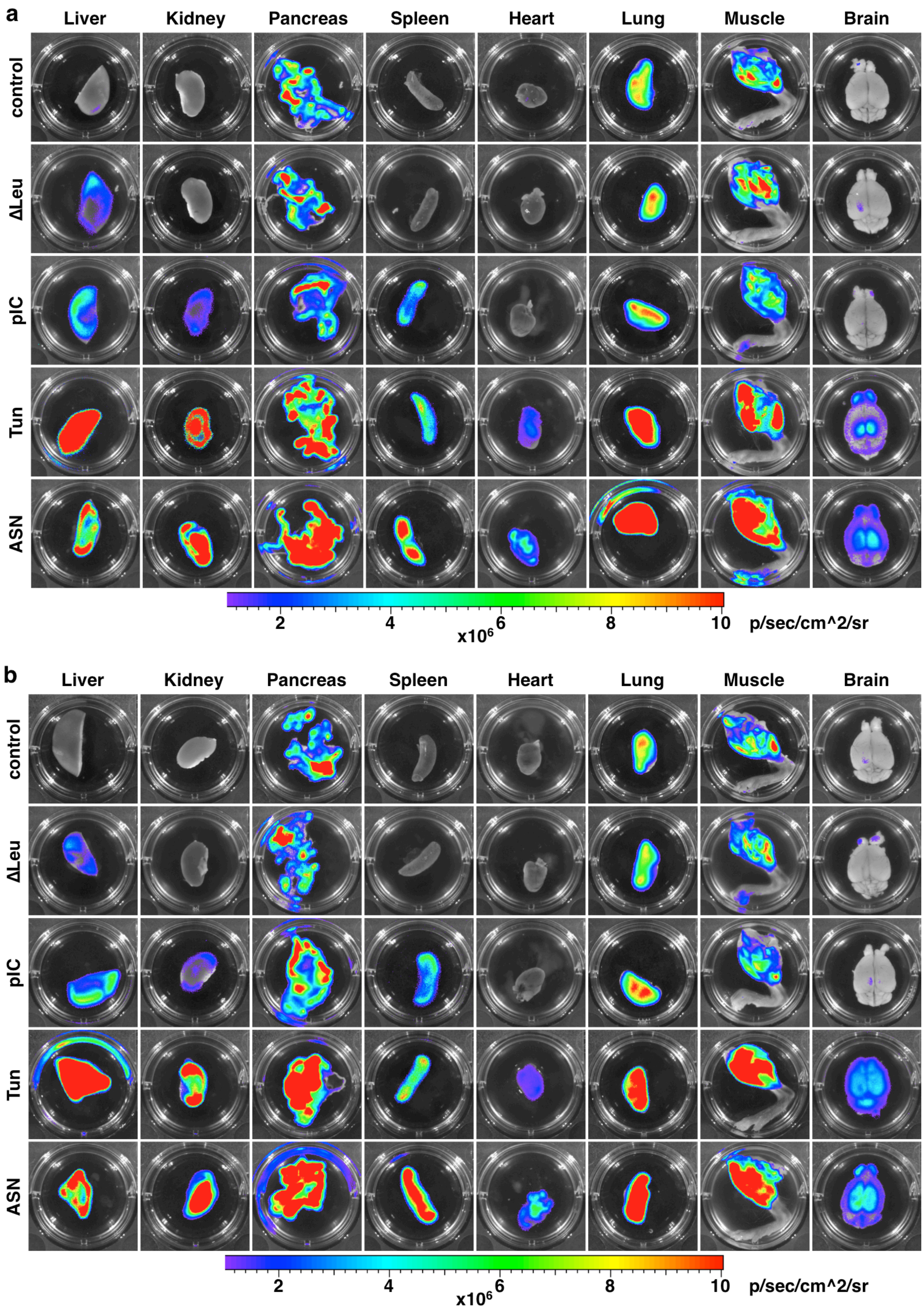

Supplementary Figure 6

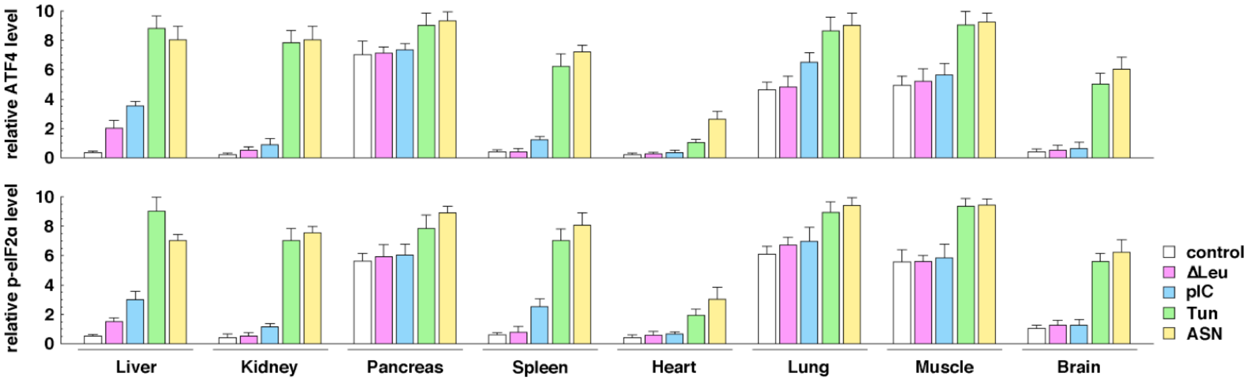

Supplementary Figure 7

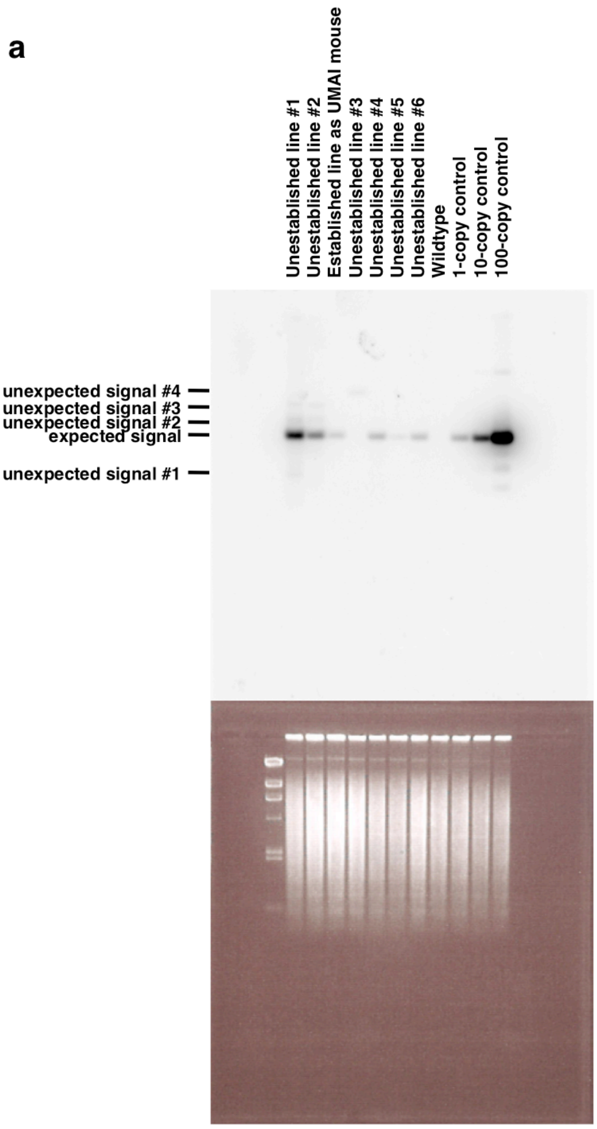

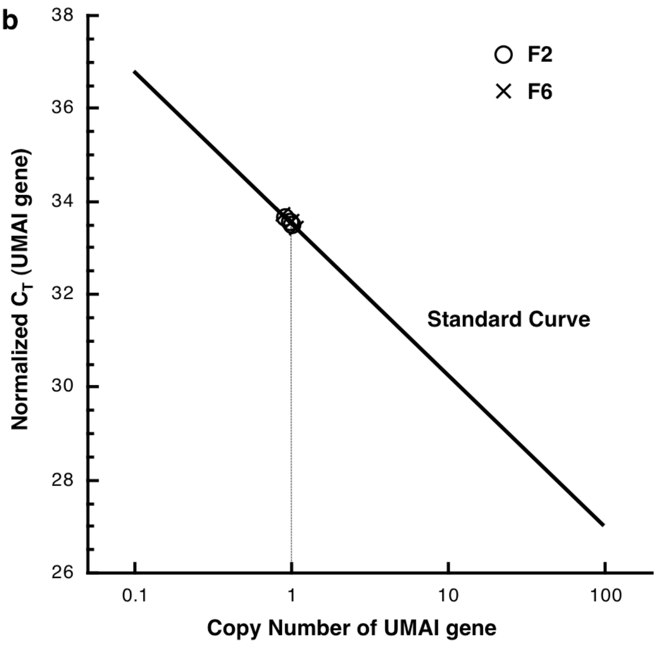

Supplementary Figure 8

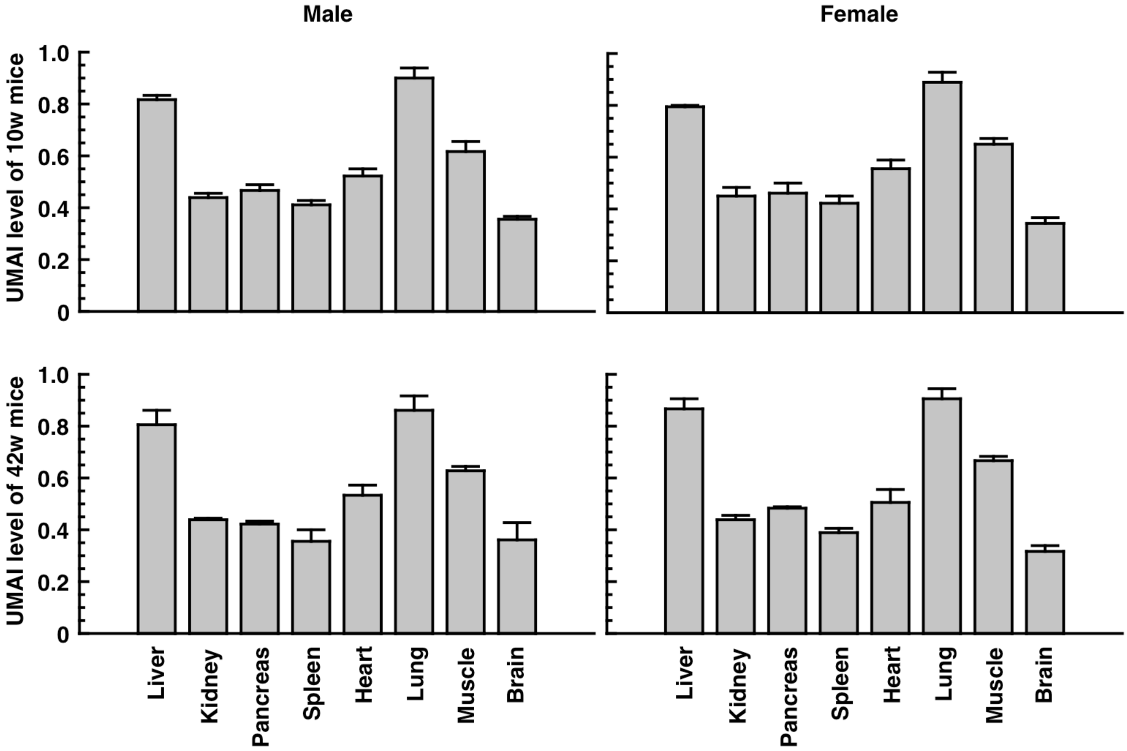

Supplementary Figure 9

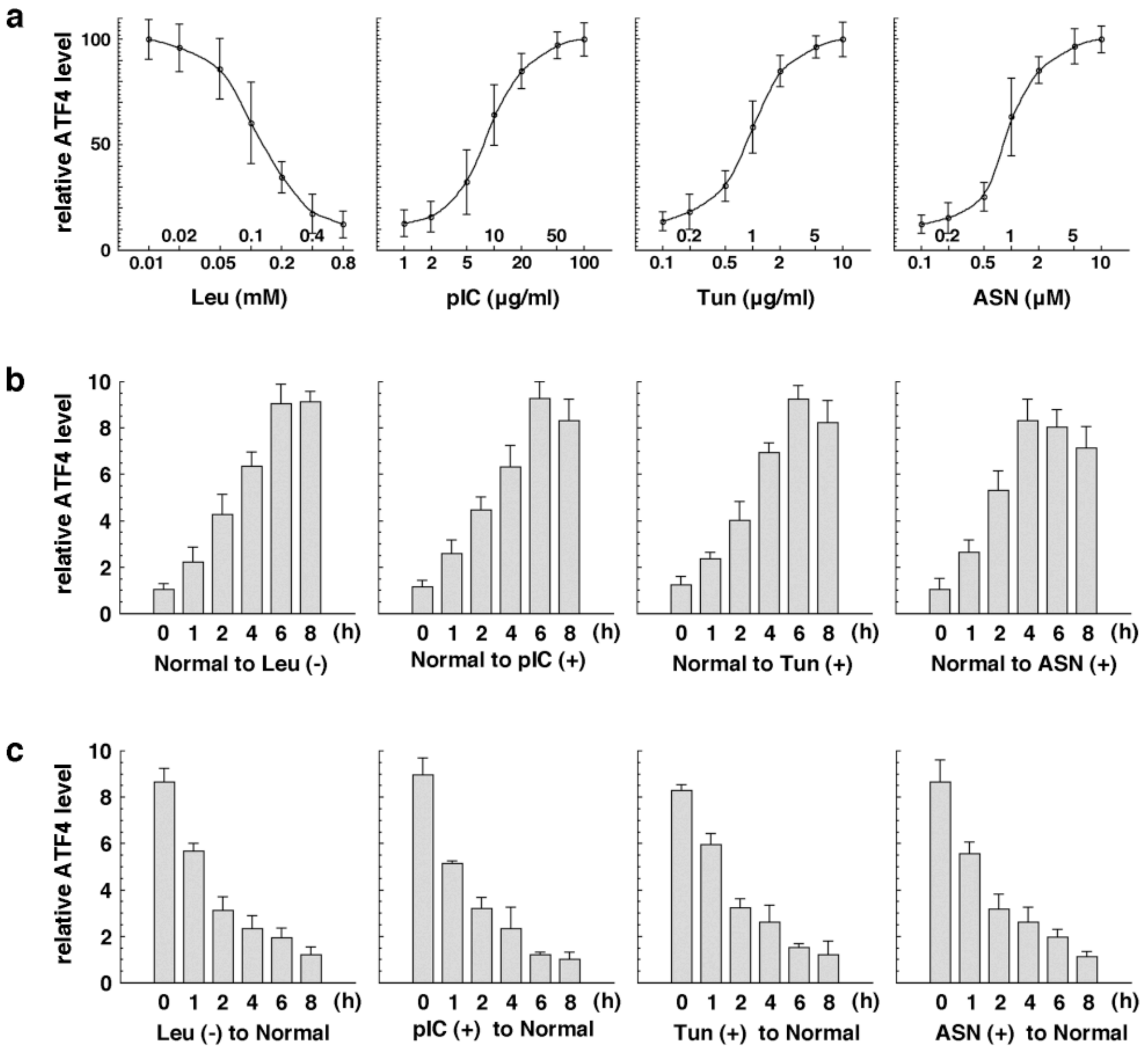

Supplementary Figure 10

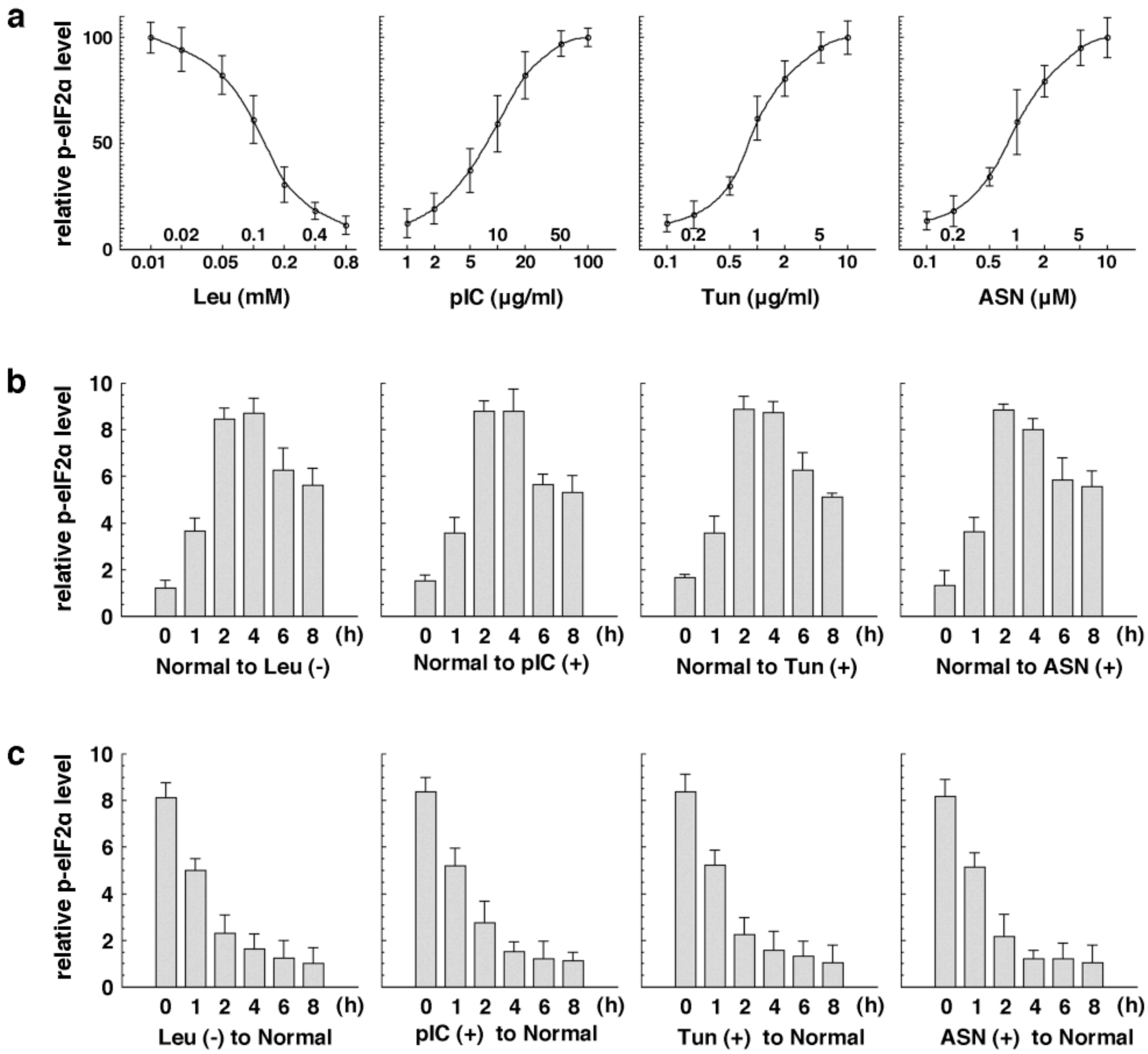

Supplementary Table

Age	Tissue	24	59	61	75	87	99	106	113	123	135	153	176	197	246	259	268	280	340
10 weeks old	Liver	2/16	0/16	1/16	1/16	1/16	0/16	0/16	1/16	0/16	0/16	0/16	2/16	1/16	0/16	0/16	0/16	0/16	0/16
	Kidney	0/16	1/16	1/16	0/16	0/16	0/16	3/16	1/16	0/16	0/16	1/16	0/16	0/16	0/16	0/16	1/16	0/16	1/16
	Pancreas	0/16	1/16	0/16	0/16	0/16	2/16	2/16	0/16	0/16	1/16	0/16	0/16	0/16	0/16	0/16	0/16	0/16	0/16
	Spleen	1/16	0/16	2/16	1/16	0/16	2/16	3/16	0/16	0/16	0/16	0/16	0/16	0/16	0/16	1/16	0/16	0/16	0/16
	Heart	2/16	2/16	0/16	0/16	1/16	0/16	3/16	1/16	1/16	0/16	0/16	1/16	0/16	0/16	0/16	0/16	0/16	0/16
	Lung	0/16	0/16	1/16	0/16	1/16	2/16	1/16	0/16	0/16	0/16	0/16	1/16	0/16	0/16	0/16	0/16	0/16	0/16
	Muscle	0/16	0/16	0/16	0/16	0/16	1/16	3/16	0/16	0/16	2/16	0/16	0/16	0/16	0/16	0/16	0/16	0/16	0/16
	Brain	1/16	1/16	0/16	1/16	0/16	2/16	1/16	0/16	0/16	0/16	1/16	0/16	0/16	0/16	0/16	1/16	0/16	0/16
42 weeks old	Liver	0/16	0/16	0/16	0/16	1/16	1/16	3/16	0/16	0/16	0/16	0/16	0/16	0/16	0/16	0/16	0/16	0/16	1/16
	Kidney	0/16	1/16	1/16	0/16	0/16	0/16	0/16	1/16	0/16	0/16	0/16	1/16	0/16	0/16	1/16	0/16	0/16	0/16
	Pancreas	0/16	0/16	0/16	1/16	0/16	0/16	3/16	1/16	0/16	0/16	0/16	0/16	0/16	0/16	0/16	0/16	0/16	0/16
	Spleen	0/16	1/16	1/16	0/16	1/16	2/16	2/16	0/16	1/16	2/16	0/16	0/16	0/16	0/16	0/16	0/16	0/16	0/16
	Heart	1/16	0/16	1/16	2/16	0/16	0/16	3/16	0/16	0/16	0/16	1/16	2/16	0/16	0/16	0/16	0/16	0/16	0/16
	Lung	2/16	2/16	0/16	0/16	0/16	2/16	1/16	1/16	0/16	0/16	0/16	0/16	0/16	0/16	0/16	0/16	0/16	0/16
	Muscle	0/16	0/16	0/16	3/16	0/16	2/16	0/16	0/16	0/16	1/16	0/16	0/16	1/16	0/16	0/16	0/16	0/16	2/16
	Brain	0/16	0/16	0/16	0/16	0/16	2/16	3/16	1/16	0/16	0/16	0/16	0/16	0/16	0/16	0/16	1/16	0/16	0/16
